# Repeated Batch Fermentation Biotechnology for the Biosynthesis of Lipid and Gamma-Linolenic Acid by *Cunninghamella bainieri* 2A1

**DOI:** 10.1155/2014/831783

**Published:** 2014-07-24

**Authors:** Marjan Ganjali Dashti, Peyman Abdeshahian, Wan Mohtar Wan Yusoff, Mohd Sahaid Kalil, Aidil Abdul Hamid

**Affiliations:** ^1^School of Biosciences and Biotechnology, Faculty of Science and Technology, Universiti Kebangsaan Malaysia, 43600 Bangi, Selangor, Malaysia; ^2^Enteric Diseases Research Cluster, Institute for Research in Molecular Medicine (INFORMM), Universiti Sains Malaysia (USM), 11800 Penang, Malaysia; ^3^Department of Chemical and Process Engineering, Faculty of Engineering and Built Environment, Universiti Kebangsaan Malaysia (National University of Malaysia), 43600 Bangi, Selangor, Malaysia

## Abstract

The biosynthesis of biomedical products including lipid and gamma-linolenic acid (GLA) by *Cunninghamella bainieri* 2A1 was studied in repeated batch fermentation. Three key process variables, namely, glucose concentration, ammonium tartrate concentration, and harvesting time, were optimized using response surface methodology. Repeated batch fermentation was carried out by the cultivation of *Cunninghamella bainieri* 2A1 in nitrogen-limited medium with various nitrogen concentration (1–4 g/L) and glucose concentration (20–40 g/L) at three time intervals (12 h, 24 h, and 48 h). Experimental results showed that the highest lipid concentration of 6.2 g/L and the highest GLA concentration of 0.4 g/L were obtained in optimum conditions, where 20.2 g/L glucose, 2.12 g/L ammonium tartrate, and 48 h harvesting time were utilized. Statistical results showed that the interaction between glucose and ammonium tartrate concentration had highly significant effects on lipid and GLA biosynthesis (*P* < 0.01). Moreover, harvesting time had a significant interaction effect with glucose and ammonium tartrate concentration on lipid production (*P* < 0.05).

## 1. Introduction

In recent years, the production of dietary lipids, including polyunsaturated fatty acids (PUFA), has received great interest from researchers. PUFA are essential fatty acids which act as precursors of many lipid-derived signaling molecules. Mammals are unable to synthesize PUFA; therefore, PUFA intake through the diet is necessary [1]. A number of PUFA have been known, such as alpha-linolenic acid, gamma-linolenic acid, eicosapentaenoic acid, and docosahexaenoic acid [1–3]. Among the variety of PUFA, gamma-linolenic acid (GLA) has merited great attentions because of its effective utilization in relieving many diseases especially multiple sclerosis. Moreover, medical intake of GLA is used in the treatment of suppressing acute and chronic inflammations. GLA is also utilized for decreasing blood cholesterol concentrations and improving atopic eczema [4].

It has been found that PUFA can be produced by fungi, zygomycetes [5]. Among zygomycetes,* Cunninghamella *species have shown to produce PUFA particularly GLA up to 20–25% of total fatty acids depending upon culture conditions [6–8]. In this regard,* Cunninghamella bainieri* 2A1 has been found to produce a large amount of GLA [9, 10]. It has also been observed that lipid production by this microorganism is affected by nitrogen source through the stress conditions created by the deficiency of nitrogen in the culture medium. Similar studies have shown that lipid synthesis by this strain is also affected by the concentration of carbon source in culture medium [10].

With advances in biotechnology, fermentation processes have been considered as a promising biotechnological method for the biosynthesis of microbial products such as food, biomedical chemicals, and pharmaceutical products. Repeated batch cultivation is a well-known method of fermentation biotechnology which is used in enhancing the productivity of microbial process. Repeated batch culture offers several advantages, including good depletion of medium in the reactor at the end of cultivation, the reuse of microbial cells for subsequent fermentation runs, high cell concentration in the culture, low time required for process operation, and process productivity. On the other hand, further studies have revealed that the repeated batch culture is affected by harvesting times of culture medium.

Response surface methodology (RSM) is a statistical method which is used for the optimization of stochastic functions and the enhancement of production process. RSM is based on the use of an experimental design particularly central composite design (CCD). This design is used for generating a statistical model of given response to fit on experimental data. RSM is also used to evaluate the effect of process parameters on the response [11].

Current study was performed to optimize three independent variables including ammonium tartrate concentration (as nitrogen source), glucose and harvesting time for improving lipid and GLA biosynthesis by* Cunninghamella bainieri* 2A1 in repeated batch fermentation biotechnology using RSM on the base of CCD.

## 2. Materials and Methods

### 2.1. Microorganism and Inoculums Preparation

Locally isolated* Cunninghamella bainieri *2A1 was obtained from the* School of Biosciences and Biotechnology, Faculty of Science and Technology, Universiti Kebangsaan Malaysia*. Stock culture was maintained on potato dextrose agar (PDA) at 4°C. Inoculum preparation was carried out using the spore suspension including 10^6^ spores/mL harvested from 7-day-old PDA plates. Seed culture was prepared by transferring 20 mL of spore suspension into 180 mL of the nitrogen-limited medium. Seed culture was then incubated at 30°C with an agitation rate of 250 rpm for 48 h and kept for the inoculation of the batch culture medium.

### 2.2. Culture Medium and Conditions

The nitrogen-limited medium employed by Kendrick and Ratledge [12] was modified and then utilized in this study with the compositions as follows (in g/L): glucose: 30; ammonium tartrate (C_4_H_12_N_2_O_6_): 1.0; KH_2_PO_4_: 7.0; Na_2_HPO_4_: 2.0; MgSO_4_ *·*7H_2_O: 1.5; CaCl_2_ *·*2H_2_O: 0.1; FeCl_3_ *·*6H_2_O: 0.008; ZnSO_4_ *·*7H_2_O: 0.0001; CuSO_4_ *·*5H_2_O: 0.001; Co (NO_3_)_2_ *·*6H_2_O: 0.0001; MnSO_4_ *·*5H_2_O: 0.0001; and yeast extract: 1.5. The pH of the medium was adjusted to 6.0 using 1.0 M HCl or 1.0 M NaOH. Repeated batch culture was carried out by continuously repeating different cycles of batch culture at same time interval. The first cycle of repeated batch culture was run by transferring 20% (v/v) of seed culture (40 mL) into 160 mL fresh medium in Erlenmeyer flasks (500 mL) to make a final 200 mL culture medium in the flask and incubating at 30°C. The second cycle of repeated batch culture was carried out by harvesting 160 mL of culture broth in the flask and adding same volume (160 mL) of fresh medium containing different concentrations of glucose and nitrogen determined (Table 1), followed by incubation at 30°C. The rest of repeated batch cycles were conducted in similar method to the second cycle. The time interval for the cycles of repeated batch culture was defined as harvesting time (hour), since at the end of this time defined volumes of culture broth were harvested. Three harvesting times were studied (Table 1). The whole repeated batch culture lasted 96 h. Harvested culture broth at the end of each cycle was tested by analytical assays and the amount of each product (biomass, lipid, GLA) was calculated by aggregating values obtained from all cycles.

### 2.3. Experimental Design

A series of experiments was designed based on a central composite design (CCD) for three independent variables, and each variable varied at three levels. According to this design the total number of experimental combinations was 2^*k*^ + 2*k* + *n*_0_, where *k* is the number of independent variables and *n*_0_ is the number of repetitions of the experiments at the centre point. This design included six star points and four replicates at the center point. The distance from the center of the design space to a star point was +1 or −1 unit. The experimental variables studied were glucose concentration, nitrogen concentration, and harvesting time. Each variable was coded at three levels of −1, 0, and +1, representing low, middle, and high level of the variables, respectively [11]. The coded values and the actual levels of the variables are given in Table 1. The design matrix of the performed experimental runs is shown in Table 2 representing eighteen treatment combinations of repeated batch culture.

### 2.4. Statistical Modeling

Experimental data from the mixture design (Table 2) were used to fit a second-order polynomial regression model (1) to represent product formation as a function of variables tested:

(1) $$\begin{array}{c} Y=a_{o}+\overset{}{\underset{}{\sum }}a_{i}X_{i}+\overset{}{\underset{}{\sum }}a_{ii}X_{i}^{2}+\overset{}{\underset{}{\sum }}a_{ij}X_{i}X_{j}, \end{array}$$

where *Y* is the measured response, *X*_*i*_ and *X*_*j*_ are the independent variables, **a**_*o*_ represents the intercept, and **a**_*i*_, **a**_*ii*_, and **a**_*ij*_ are the regression coefficients of the model [13]. The behavior of the generated model for three independent variables was expressed mathematically as follows:

(2) Y=ao+a1X1+a2X2+a3X3+a11X12+a22X22+a33X32+a12X1X2+a13X1X3+a23X2X3,

where *Y* is the measured response (lipid and GLA), **a**_1_, **a**_2_, and **a**_3_ are linear coefficients, **a**_11_, **a**_22_, and **a**_33_ are squared coefficients, and **a**_12_, **a**_13_, and **a**_23_ are interaction coefficients. *X*_1_, *X*_2_, and *X*_3_ represent coded values of glucose concentration (g/L), ammonium tartrate (g/L), and harvesting time (h), respectively. Statistical analysis of the data was performed using Design-Expert software (version 6.0.6 Stat-Ease, Inc.). The same software was used for numerical optimization. Numerical optimization was used for simultaneous optimization of the multiple responses in which all the independent variables were kept within the range determined, while the responses were maximized.

### 2.5. Analytical Methods

The fungal mycelium was harvested by the filtration of 100 mL of culture suspension using filter paper (Whatman number 1). The filtered mycelium was washed with 200 mL of distilled water, stored at −20°C for 24 h and then put under freeze-dried conditions (Shell Freeze Dry, LABCONCO LYPH.LOCK6) for 24 h to obtain the dry weight. The dry weight of fungal cells was determined using a balance (AND GR-200). The dry weight of cells was used to determine the biomass and lipid concentration as well as lipid content. Dried mycelia were then ground using a pestle and mortar, followed by lipid extraction. Lipid was extracted by filtration of a mixture of chloroform and methanol in a ratio of 2 : 1 (v/v). The filtrate was washed with 150 mL of NaCl (1% w/v), followed by an addition of 150 mL of distilled water [14]. The chloroform layer was obtained and evaporated using rotary evaporator (BUCHI Rotavapor R-124). Lipid residues were dissolved in a minimal amount of diethyl ether and transferred to a vial. Extracted lipid was sent to Analytical Services Laboratory Malaysian Coco board (PusatInovasi and Teknologi Koko, lot Pt 12621, Kawasan Preindustrian Nilai, 71800 Nilai, Negeri Sembilan Darul Khusus) to determine gamma-linolenic acid (GLA) concentration. The ratio of initial carbon content to nitrogen content (C/N) used in the medium was calculated based on the total mol of carbon content obtained from glucose, ammonium tartrate, and yeast extract (assuming 10% w/w of yeast extract is composed of carbon content) divided to total mol of nitrogen content obtained from ammonium tartrate and yeast extract (assuming 8.9% w/w of yeast extract is composed of nitrogen content).

## 3. Results and Discussion

### 3.1. Biomass Production

Experimental results of biomass production according to CCD are shown in Table 2. As can be observed, three independent variables (glucose concentration, ammonium tartrate concentration, and harvesting time) were controlled at the levels determined by experimental design, which were represented as −1, 0, and +1 (Table 1). As can be seen from results in Table 2, treatments 1, 10, 11, 13, and 14 showed high levels of biomass concentration. Treatment 4, 10, 11, and 13 included center points of design in which same glucose concentration, ammonium tartrate concentration, and harvesting time were used for the estimation of test error. By applying multiple regression analysis to the test results, a second-order polynomial equation (3) was derived to represent biomass as a function of glucose concentration, nitrogen concentration, and harvesting time:

(3) Y=20.73+0.19X1+1.70X2+0.04X3−0.72X12−2.37X22+0.83X32+0.26X1X2−0.21X1X3+0.51X2X3,

where *Y* is the measured biomass (g/L). The statistical significance of the fitted model was evaluated using the analysis of variance (ANOVA) for biomass production (Table 3). As the results shown in Table 3, calculated model's *F* value of 22.88 with a probability value (Prob > *F*) less than 0.0001 suggested that the selected quadratic model was significant and fitted well to the experimental data (*P* < 0.01). The lack of fit is a measure of the failure of a model to represent data in the experimental domain at which data points were not included in the regression model or variations in the models cannot be accounted by random error. If there is a significant lack of fit, the response is not fitted. The *F* value for lack of fit with a value of 6.0 implied that the lack of fit was insignificant and hence the model was valid for further studies. It is evident from Table 3 that biomass production was influenced by significant effects of ammonium tartrate concentration as linear term (*X*_2_) and quadratic term (*X*_2_^2^) indicating this point that the changes of this nutrient in culture could drastically affect the biomass production (*P* < 0.01). Moreover, the quadratic term of harvesting time (*X*_3_^2^) showed significant effect on biomass concentration at 95% probability level (*P* < 0.05). Statistical results also revealed that the interaction effect between ammonium tartrate concentration and the harvesting time of culture (*X*_2_ *X*_3_) were found significant at 95% probability level (*P* < 0.05).

**Figure 1:**
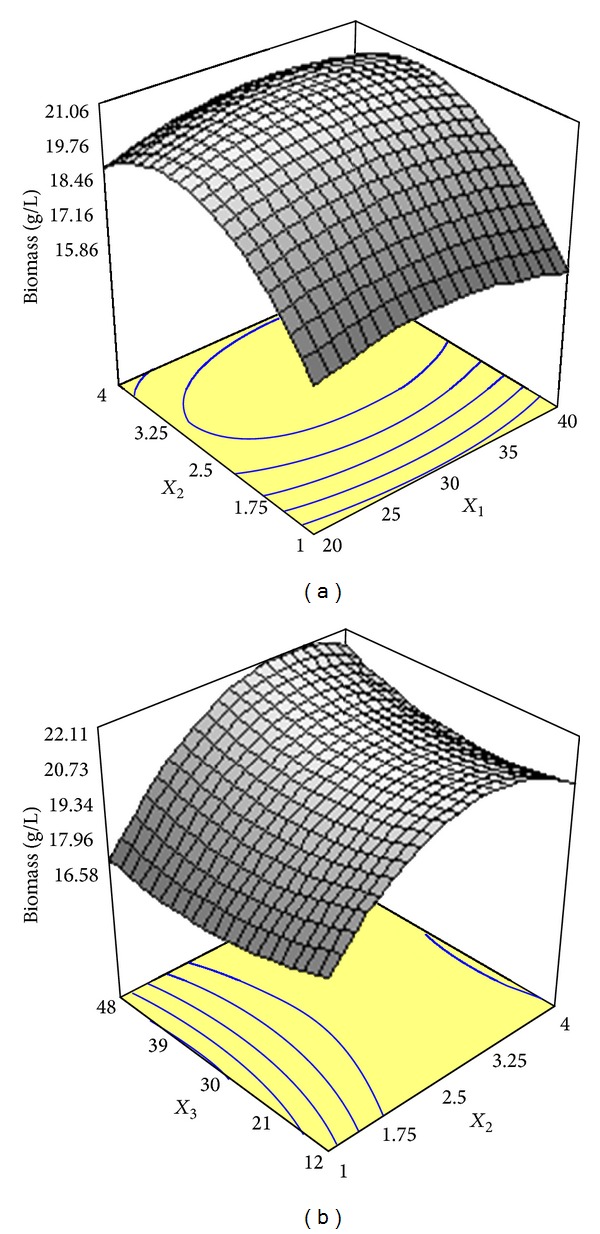
(a) Response surface plot showing the simultaneous effects of glucose concentration (*X*_1_) and ammonium tartrate concentration (*X*_2_) on biomass synthesis by* Cunninghamella bainieri* 2A1 in repeated batch fermentation at 30°C for 96 h. (b) Response surface plot showing the simultaneous effects of ammonium tartrate concentration (*X*_2_) and harvesting time (*X*_3_) on biomass synthesis by* Cunninghamella bainieri* 2A1 in repeated batch fermentation at 30°C for 96 h.

The multiple coefficient of determination (*R* ^2^) represents the variability in the values of the formed response which can be explained by the test factors and their interactions. In this case, *R* ^2^ value for biomass concentration was high enough (0.9626), indicating that 96.26% data variability could be explained by the quadratic model and only 3.74% of total variability in the response could not be explained by the regression model (Table 3).  Figure 1(a) shows the simultaneous effects of glucose concentration and ammonium tartrate concentration on the biomass synthesis. As can be seen, an increase in biomass concentration occurred when glucose concentration began to increase with the low level of ammonium tartrate. A subsequent rise in ammonium tartrate concentration resulted in a significant increase in biomass concentration which indicated the significant effect of ammonium tartrate concentration on biomass production compared to glucose concentration.  Figure 1(b) shows the interaction effects between ammonium tartrate concentration and harvesting time on the biomass production. As can be seen, increasing harvesting time from 12 h to 30 h (center point) decreased biomass concentration by* Cunninghamella bainieri* 2A1, while an increment in harvesting time from 30 h to 48 h increased biomass synthesis. On the other hand, a rise in ammonium tartrate concentration from 1 g/L to optimum levels caused an exponential increase of biomass. In line with this study, Fakas et al. [15] tested different concentrations of nitrogen in culture medium of* Cunninghamella echinulata* grown on complex organic nitrogen sources. They observed that increasing concentration of nitrogen triggered higher biomass production. In this regard, Vamvakaki et al. [16] noted that a supplementation of ammonium sulfate to cheese whey favored the consumption of carbon source (lactose) by two zygomycetes, namely,* Thamnidium elegans* and* Mucor* sp., which in turn increased the production of biomass.

### 3.2. Lipid Synthesis

Table 2 shows test results of lipid production by* Cunninghamella bainieri* 2A1 at varied combinations of glucose concentration, ammonium tartrate concentration, and harvesting time according to CCD. As shown in this table, treatment 1, 3, 12, and 14 revealed the high levels of lipid concentration. A second-order polynomial model was generated to represent the production of lipid concentration in repeated batch process by a mathematical equation as follows:

(4) Y=4.55+0.051X1−0.15X2+1.28X3−0.18X12−0.35X22+0.098X32+0.26X1X2−0.11X1X3−0.12X2X3,

where *Y* is the lipid production (g/L).  Table 4 shows the significance of linear, interaction, and quadratic effects of the variables based on their probability values. As can be observed from Table 4, computed model's *F* value of 134.41 with a probability value (Prob > *F*) of less than 0.0001 indicated that the quadratic regression model was significantly fitted on experimental data (*P* < 0.01). The lack of fit related to *F* value (1.82) implied that the lack of fit was not significant. Hence, the regression model was acceptable for further evaluation.

As shown in Table 4, the linear effect of ammonium tartrate concentration and harvesting time (*X*_2_ and *X*_3_) were found significant at 99% probability level (*P* < 0.01). Moreover, the quadratic term of glucose and ammonium tartrate concentration (*X*_1_^2^ and *X*_2_^2^) had significant effect on lipid concentration at 95% and 99% probability levels, respectively. The statistical model also showed that the interaction effect between all variables studied were significant (Table 4). The multiple coefficient of determination (*R* ^2^) with a satisfactory value of 0.9934 implied that 99.34% of the variability in the response could be attributed to the independent parameters studied (glucose concentration, ammonium tartrate concentration, and harvesting time) and only 0.66% of the total variation could not be explained by the model.

**Figure 2:**
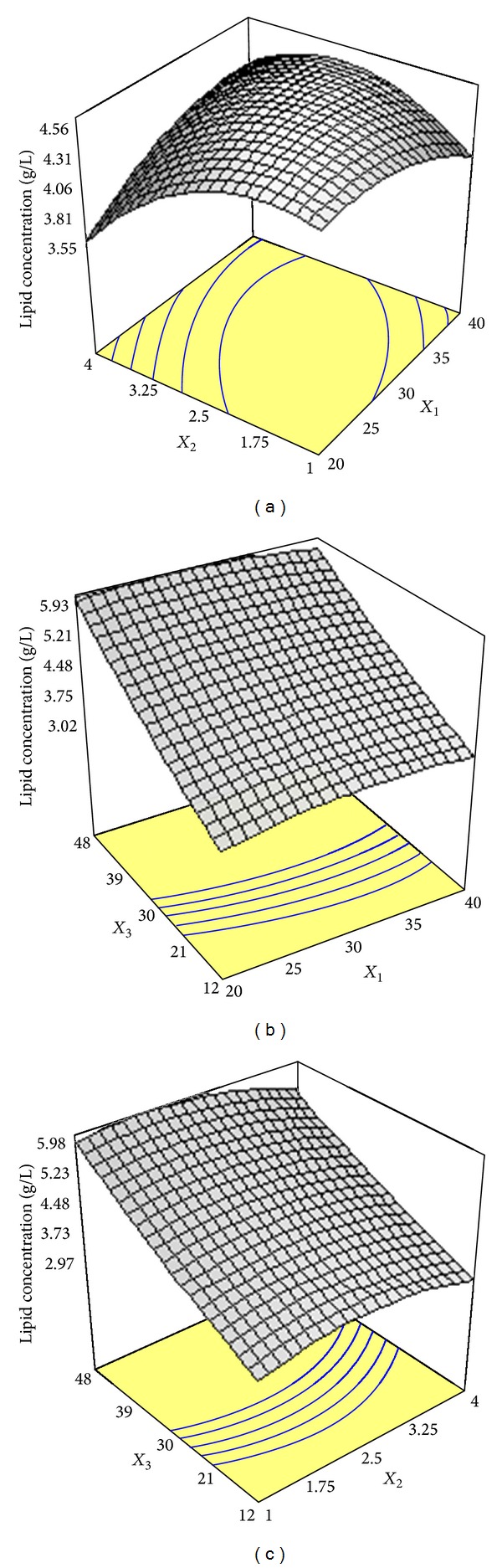
(a) Response surface plot showing the simultaneous effects of glucose concentration (*X*_1_) and ammonium tartrate concentration (*X*_2_) on lipid biosynthesis by* Cunninghamella bainieri* 2A1 in repeated batch fermentation at 30°C for 96 h. (b) Response surface plot showing the simultaneous effects of glucose concentration (*X*_1_) and harvesting time (*X*_3_) on lipid biosynthesis by* Cunninghamella bainieri* 2A1 in repeated batch fermentation at 30°C for 96 h. (c) Response surface plot showing the simultaneous effects of ammonium tartrate concentration (*X*_2_) and harvesting time (*X*_3_) on lipid biosynthesis by* Cunninghamella bainieri* 2A1 in repeated batch fermentation at 30°C for 96 h.

Figure 2(a) shows the concurrent effects of glucose concentration and ammonium tartrate concentration on the lipid production. Following the rise in glucose concentration resulted in quadratic rise in the level of lipid concentration. Similarly, an increase in ammonium tartrate up to optimum level concomitantly increased lipid production.  Figure 2(b) illustrates the interaction effect between glucose concentration and harvesting time. As can be found, variations in glucose concentration led to an exponential increase in response, while linear increase in lipid was obtained when harvesting time increased from 12 h to 48 h. Similar pattern was observed for simultaneous effect of ammonium tartrate concentration and harvesting time on lipid concentration as shown in Figure 2(c).

The values obtained for lipid content (lipid concentration/biomass concentration × 100) based on CCD are shown in Table 2. By applying multiple regression analysis to the test results, a second-order polynomial equation (5) was obtained in order to represent lipid content as a function of glucose concentration, ammonium tartrate concentration, and harvesting time.

**Figure 3:**
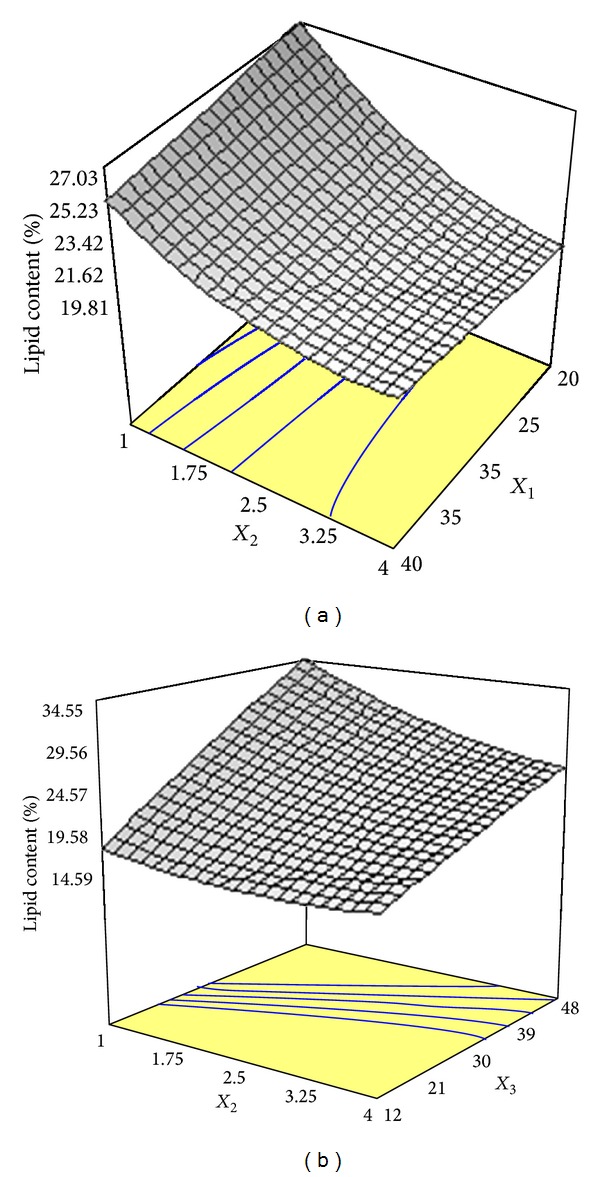
(a) Response surface plot showing the simultaneous effects of glucose concentration (*X*_1_) and ammonium tartrate concentration (*X*_2_) on lipid content produced by* Cunninghamella bainieri* 2A1 in repeated batch fermentation at 30°C for 96 h. (b) Response surface plot showing the simultaneous effects of ammonium tartrate concentration (*X*_2_) and harvesting time (*X*_3_) on lipid content produced by* Cunninghamella bainieri* 2A1 in repeated batch fermentation at 30°C for 96 h.

(5) Y=21.84−0.15X1−2.86X2+6.74X3+0.042X12+1.39X22−0.41X32+0.75X1X2−0.15X1X3−2.12X2X3,

where *Y* is the measured lipid content (%). The significance of the response surface quadratic model was evaluated using statistical analysis of variance (Table 5). Obviously, the model *F* value of 276.48 with a probability value (Prob > *F*) less than 0.0001 implied that the regression model was significant (*P* < 0.01). Furthermore, the lack of fit of the model with the value of 5.41 indicated that the lack of fit was insignificant. Therefore, the empirical model adequately fitted on the experimental results. As shown in Table 5, linear effect of ammonium tartrate concentration (*X*_2_) and harvesting time (*X*_3_) was significant at 99% probability level (*P* < 0.01). Obviously, quadratic terms of ammonium tartrate concentration (*X*_2_^2^) also had highly significant effect on lipid content at 99% probability level (*P* < 0.01). It was also observed that the interaction effect between glucose concentration and ammonium tartrate concentration (*X*_1_ *X*_2_) and the interaction effect between ammonium tartrate concentration and harvesting time (*X*_1_ *X*_2_) on lipid content were highly significant (*P* < 0.01). The multiple coefficient of determination (*R* ^2^) with an acceptable value of 0.9968 indicated that 99.68% of the variability in response could be explained by the model.  Figure 3(a) shows the influences of glucose concentration and ammonium tartrate concentration on the lipid content. As can be seen, variations in ammonium tartrate concentration had no effect on the glucose concentration range of 20 to 40 g/L, indicating the lack of interaction between these variables.  Figure 3(b) illustrates simultaneous effects of ammonium tartrate concentration and harvesting time on the lipid content. It is evident that an increase in response was obtained when ammonium tartrate concentration and harvesting time concurrently affected product formation by* Cunninghamella bainieri* 2A1 in the range of tested levels.

Many studies have already been carried out to find the pivotal roles of glucose and nitrogen source in the mechanism of lipid accumulation by oleaginous microorganisms [17, 18]. It has been found that nitrogen limitation in culture medium acts as a stimulating factor for microbial lipid biosynthesis by oleaginous microorganisms. Lipid accumulation in oleaginous microorganisms is carried out by cell nitrogen depletion, while glucose continues to be assimilated by the oleaginous microorganisms to form lipids. During the lipid synthesis phase, the proportion of lipid is high; however, it decreases when a decrease in biomass production occurs [19]. This study showed that an increase in glucose concentration and nitrogen source drastically affected lipid production using different C/N molar ratios. An increase in ammonium tartrate higher than optimum concentration decreased lipid production (Figures 2(a), 2(b), and 2(c)). This finding was consistent with the results reported by Fakas et al. [15] who observed that excessive nitrogen concentration in culture medium decreased lipid synthesis by* Cunninghamella echinulata*. As can be seen from Table 2, increasing in C/N ratio showed generally higher lipid concentration and lipid content at similar fermentation time. Similarly, Economou et al. [20] observed that a rise in the concentrations of carbon and nitrogen under increased C/N values constantly increased lipid production by* Mortierella isabellina* using rice hulls hydrolysate as substrate. The positive effect of initial glucose concentration on lipid production could be attributed to the fact that increased glucose provides higher carbon and energy source with enhanced C/N molar ratio which leads to a rise in biomass production and higher lipid accumulation [21]. On the other hand, increased cultivation time favored lipid accumulation and lipid percentage with the similar value of C/N ratio. Similar trend was reported by Gema et al. [6] who cultivated* Cunninghamella echinulata* on glucose as a carbon source. They observed that a rise in C/N ratio from 94 to 163 under increased incubation time resulted in an increment in lipid synthesis and lipid content to 4.4 g/L and 16.4% after 480 h. However, increasing fermentation time could have adverse effect on lipid concentration as Fakas et al. [22] observed that the cultivation of* Cunninghamella echinulata* on a tomato waste resulted in the highest lipid production with the value of 7.8 g/L, followed by a decrease in lipid concentration to 4.4 g/L when fermentation further proceeded. The three-dimensional response surface graphs plotted in Figures 2(b) and 2(c) indicated that a rise in lipid production was consistent with increased harvesting time from 12 to 48 h. It has been found that there is a reciprocal relation between process time and lipid production in oleaginous fungi. Tao and Zhang [23] showed that* Cunninghamella echinulata* mainly used glucose to produce lipid up to a maximum level at 96 h of batch culture. In contrast, Papanikolaou et al. [24] reported that the highest lipid level was achieved after 310–400 h of cultivation time. The three-dimensional response surface graph plotted in Figure 3(b) indicated that a decrease in nitrogen source had a pivotal effect on forming high lipid content as harvesting time increased from 12 h to 48 h. Fakas et al. [25] reported the highest lipid content (12%) was produced by* Mortierella isabellina* ATHUM 2935 on pear pomac at increased fermentation time of 212 h after inoculation.

### 3.3. GLA Biosynthesis

Table 2 shows the experimental results of GLA production obtained from the cultivation of* C. bainieri* 2A1 in different levels of glucose concentration, ammonium tartrate concentration, and harvesting time determined by the experimental design. As shown in Table 2, treatments 6, 7, and 12 revealed the high levels of GLA concentration. A second-order polynomial model was constructed to represent the biosynthesis of GLA in repeated batch fermentation biotechnology by a mathematical equation as follows:

(6) Y=0.18−0.047X1+0.025X2−0.022X3+7.976E−003X12−0.012X22+0.13X32+0.071X1X2−3.750E−003X1X3−0.014X2X3,

where *Y* is the amount of GLA production.  Table 6 represents the analysis of variance (ANOVA) for GLA concentration obtained. As can be found, the model *F* value of 8.44 with a probability value (Prob > *F*) of 0.0031 implied that the regression model was significant (*P* < 0.01). Furthermore, the lack of fit of the model with the value of 4.52 indicated that the lack of fit was insignificant. Therefore, the empirical model adequately fitted on the experimental results.

**Figure 4:**
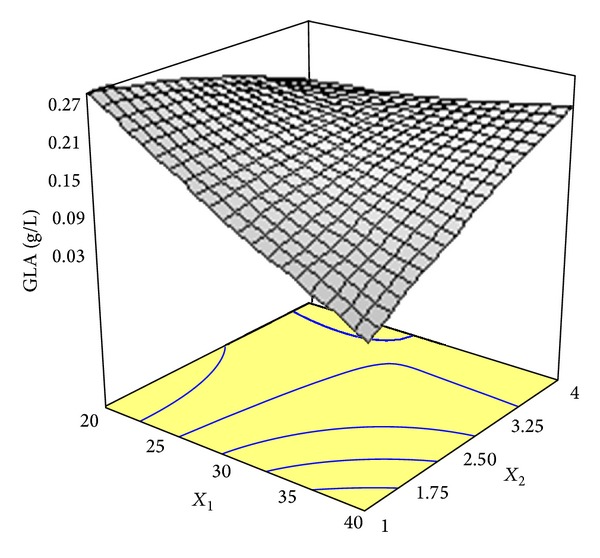
Response surface plot showing the simultaneous effects of glucose concentration (*X*_1_) and ammonium tartrate concentration (*X*_2_) on GLA biosynthesis by* Cunninghamella bainieri* 2A1 in repeated batch fermentation at 30°C for 96 h.

As shown in Table 6, the quadratic term of harvesting time (*X*_3_^2^) and the interaction effect between glucose concentration and ammonium tartrate concentration (*X*_1_ *X* ^2^) had highly significant effects on GLA production (*P* < 0.01). The statistical model also showed that the linear effect of glucose concentration (*X*_1_) on GLA biosynthesis was significant (*P* < 0.05). The multiple coefficient of determination (*R* ^2^) with a satisfactory value of 0.9047 implied that the regression model could explain 90.47% of the variability in the response (GLA production) and only 9.53% of the total variation could not be explained by the regression model. The three-dimensional response surface graph of simultaneous effect of glucose concentration and ammonium tartrate concentration on GLA synthesis was constructed to illustrate the interaction effect between these variables (Figure 4). As can be observed, a marked rise in the response was formed with increased nitrogen source concentrations in the range of levels tested. However, increasing glucose concentration brought about a drop in GLA synthesis. Hence, variations in response were affected by different concentrations of glucose and ammonium tartrate, indicating that the GLA production was influenced by interaction between these parameters.

As mentioned previously, ammonium tartrate ameliorated GLA production (Figure 4). Similar trend was reported by Gema et al. [6] who observed that an addition of supplementary amount of ammonium sulfate to orange peel enhanced GLA synthesis.

Current study showed that increasing fermentation time from 12 h to 30 h reduced GLA production, while further increase in cultivation time from 30 h to 48 h led to an increment in GLA concentration (Table 2). It has been found that GLA production is affected by fermentation time so that high GLA production occurs at early cultivation time in which lipid production is in a low rate. Variations in GLA content was studied by Fakas et al. [28] who measured various GLA content during growth of* Cunninghamella echinulata* on sugar-based substrate, where high GLA content was detected at early lipid production process. However, GLA content start dwindling as fermentation proceeds in which lipid content is enhanced, followed by an increased GLA synthesis at the late fermentation time where lipid accumulation process wanes [7, 21].

### 3.4. Fatty Acid Composition of Total Lipid Produced by* C. bainieri* 2A1

Total lipid produced by* C. bainieri* 2A1 in different fermentation runs conducted was analyzed to determine its fatty acid composition.  Table 7 shows the amount of fatty acids measured in each trail. As can be seen, the main fatty acid produced was oleic acid (^Δ9^C18:1) followed by stearic acid (C18:0) and palmitic acid (C16:0), while linoleic acid (^Δ9,12^C18:2) and GLA (^Δ6,9,12^C18:3) were in lower concentrations. As sown in Table 7, the maximum concentration of oleic acid (2.36 g/L), stearic acid (1.26 g/L), palmitic acid (1.63 g/L), and linoleic acid (0.98) was produced in fermentation runs of 18, 1, 7, and 1, respectively, with a relatively low C/N ratio, indicating that increased nitrogen source favored the biosynthesis of these fatty acids. Similarly, Economou et al. [17] found that the oleic acid was predominant fatty acid produced by* Mortierella isabellina* grown on rice hulls, which was followed by palmitic acid and linoleic acid that were measured in high amounts. Zikou et al. [26] also found that oleic acid was the highest fatty acid among fatty acids detected in lipid produced by* Thamnidium elegans* cultivated on glucose and xylose-based media, which was followed by palmitic acid, linoleic acid, and GLA in significant quantities.

### 3.5. Determination of Optimum Conditions and Validation of the Regression Model

By an analysis of the quadratic models using Design-Expert software, optimum conditions for attaining the highest production of biomass, lipid, and GLA concentration and also lipid content were obtained. The analytical results indicated that the optimal levels of glucose concentration, ammonium tartrate concentration, and harvesting time were 20.2 g/L, 2.12 g/L, and 48 h, respectively. The statistical analysis predicted that under optimum conditions the values of 20.25 g/L, 5.92 g/L, 0.35 g/L, and 30.06% could be achieved for biomass concentration, lipid concentration, GLA concentration, and lipid content, respectively. In order to verify the accuracy of the statistical model,* Cunninghamella bainieri* 2A1 was cultivated under optimum conditions in repeated batch culture. Test results revealed that the values of 20.55 g/L, 6.2 g/L, 0.4 g/L, and 30.24% were produced for biomass concentration, lipid concentration, GLA concentration, and lipid content, respectively. The closeness of the values in the verification experiment and predicted values by the model confirmed the accuracy of the selected model and reproducibility of the responses.  Table 8 enumerates lipid and GLA synthesis by oleaginous zygomycetes reported in previous studies in comparison to the current study.

## 4. Conclusion

This study evaluated the feasibility of repeated batch fermentation biotechnology for lipid and GLA synthesis by* Cunninghamella bainieri* 2A1. Process optimization of key operating parameters, namely, glucose concentration, ammonium tartrate concentration and harvesting time, was successfully carried out using RSM based on CCD. This study revealed that a low ammonium tartrate and glucose concentration were required in both high lipid and GLA synthesis. However, high concentrations of these variables only lead to high biomass concentration. This finding suggests that repeated batch fermentation can be considered as a promising biotechnological method for large-scale biosynthesis of lipid and GLA in the context of biomedical products development.

## Figures and Tables

**Table 1:** Test variables and levels for central composite design (CCD).

Variable	Symbol	Level					
		Actual range			Coded value		
		Low	Middle	High	Low	Middle	High
Glucose concentration (g/L)	*X*_1_	20	30	40	−1	0	+1
Ammonium tartrate (g/L)	*X*_2_	1	2.5	4	−1	0	+1
Harvesting time (h)	*X*_3_	12	30	48	−1	0	+1

**Table 2:** Central composite design and experimental results for the biomass concentration, lipid concentration, lipid content, and GLA concentration produced by *Cunninghamella bainieri* 2A1 in repeated batch fermentation at 30°C for 96 h.

Run	*X*_1_	*X*_2_	*X*_3_	C/N	Biomass (g/L)	Lipid concentration (g/L)	Lipid content (%)	GLA (g/L)
1	0	0	+1	29.45	21.5	6.1	28.3	0.33
2	+1	−1	−1	71.5	17.65	2.85	16.1	0.17
3	+1	−1	+1	71.5	15.95	5.35	33.5	0.15
4	0	0	0	29.45	20.9	4.5	21.5	0.21
5	0	+1	0	20.38	19.6	4.1	20.9	0.22
6	−1	−1	−1	35	17.15	3.0	17.4	0.45
7	+1	+1	−1	27.5	20.4	3.26	15.9	0.40
8	−1	0	0	19.45	19.5	4.4	22.5	0.18
9	0	0	−1	29.45	20.8	3.2	15.3	0.30
10	0	0	0	29.45	21.0	4.5	21.4	0.19
11	0	0	0	29.45	21.5	4.7	21.8	0.15
12	−1	−1	+1	35	16.65	5.95	35.7	0.40
13	0	0	0	25.1	21.15	4.5	21.2	0.18
14	+1	+1	+1	27.5	21.1	5.3	25.1	0.28
15	−1	+1	−1	13.46	19.2	2.4	14.5	0.35
16	0	−1	0	53	16.3	4.3	26.3	0.12
17	+1	0	0	39.73	19.7	4.35	22.0	0.20
18	−1	+1	+1	13.46	20.4	4.85	24.0	0.29

*X*_1_: glucose concentration (g/L); *X*_2_: ammonium tartrate (g/L); *X*_3_: harvesting time (h); C/N: the ratio of initial carbon content (mol) to nitrogen content (mol) used in medium.

**Table 3:** Analysis of variance for the second-order polynomial model of biomass synthesis by *Cunninghamella bainieri* 2A1 using repeated batch cultivation at 30°C for 96 h.

Source	Polynomial coefficients	Sum of squares	DF	Mean square	*F* value	Prob > *F*
Model		58.56	9	6.51	22.88	<0.0001∗∗
Intercept	20.73					
*X*_1_	0.19	0.36	1	0.36	1.27	0.2926
*X*_2_	1.70	28.90	1	28.90	101.61	<0.0001∗∗
*X*_3_	0.040	0.016	1	0.016	0.056	0.8185
*X*_1_^2^	−0.72	1.42	1	1.42	4.98	0.0561
*X*_2_^2^	−2.37	15.26	1	15.26	53.66	<0.0001∗∗
*X*_3_^2^	0.83	1.85	1	1.85	6.51	0.0341∗
*X*_1_ *X*_2_	0.26	0.55	1	0.55	1.94	0.2014
*X*_1_ *X*_3_	−0.21	0.36	1	0.36	1.27	0.2924
*X*_2_ *X*_3_	0.51	2.10	1	2.10	7.39	0.0263∗
Residuals		2.28	8	0.28		
Lack of fit		2.07	5	0.41	6.0	0.0856
Pure error		0.21	3	0.069		

*Statistically significant at 95% probability level.

**Statistically significant at 99% of probability level.

*X*_1_: glucose concentration (g/L); *X*_2_: ammonium tartrate (g/L); *X*_3_: harvesting time (h). *X*_1_^2^, *X*_2_^2^, and *X*_3_^2^: the quadratic terms; *X*_1_ *X*_2_,  *X*_1_ *X*_3_, and *X*_2_ *X*_3_: the interaction terms.

*R* ^2^ = 0.9626.

**Table 4:** Analysis of variance for the second-order polynomial model of lipid synthesis by *Cunninghamella bainieri* 2A1 using repeated batch cultivation at 30°C for 96 h.

Source	Polynomial coefficients	Sum of squares	DF	Mean square	*F* value	Prob > *F*
Model		18.27	9	2.03	134.41	<0.0001∗∗
Intercept	4.55					
*X*_1_	0.051	0.026	1	0.026	1.72	0.2258
*X*_2_	−0.15	0.24	1	0.24	15.70	0.0042∗∗
*X*_3_	1.28	16.49	1	16.49	1091.56	<0.0001∗∗
*X*_1_^2^	−0.18	0.085	1	0.085	5.61	0.0453∗
*X*_2_^2^	−0.35	0.34	1	0.34	22.22	0.0015∗∗
*X*_3_^2^	0.098	0.026	1	0.026	1.73	0.2253
*X*_1_ *X*_2_	0.26	0.53	1	0.53	35.12	0.0004∗∗
*X*_1_ *X*_3_	−0.11	0.092	1	0.092	6.12	0.0385∗
*X*_2_ *X*_3_	−0.12	0.12	1	0.12	7.63	0.0246∗
Residuals		0.12	8	0.015		
Lack of fit		0.091	5	0.018	1.82	0.3305
Pure error		0.030	3	1.000*E* − 002		

*Statistically significant at 95% probability level.

**Statistically significant at 99% of probability level.

*X*_1_: glucose concentration (g/L); *X*_2_: ammonium tartrate (g/L); *X*_3_: harvesting time (h). *X*_1_^2^, *X*_2_^2^, and *X*_3_^2^: the quadratic terms; *X*_1_ *X*_2_, *X*_1_ *X*_3_, and *X*_2_ *X*_3_: the interaction terms.

*R* ^2^ = 0.9934.

**Table 5:** Analysis of variance for the second-order polynomial model of lipid content produced by *Cuninghamella bainieri* 2A1 using repeated batch cultivation at 30°C for 96 h.

Source	Polynomial coefficients	Sum of squares	DF	Mean square	F value	Prob > F
Model		583.88	9	64.88	276.48	<0.0001∗∗
Intercept	21.84					
* *X_1_	−0.15	0.23	1	0.23	0.96	0.3561
X_2_	−2.86	81.80	1	81.80	348.59	<0.0001∗∗
X_3_	6.74	454.28	1	454.28	1936.01	<0.0001∗∗
*X*_1_^2^	0.042	4.704E − 003	1	4.704E − 003	0.020	0.8909
*X*_2_^2^	1.39	5.25	1	5.25	22.37	0.0015∗∗
*X*_3_^2^	−0.41	0.45	1	0.45	1.93	0.2027
X_1_X_2_	0.75	4.50	1	4.50	19.18	0.0024∗∗
X_1_X_3_	−0.15	0.18	1	0.18	0.77	0.4066
X_2_X_3_	−2.12	36.13	1	36.13	153.96	<0.0001∗∗
Residuals		1.88	8	0.23		
Lack of fit		1.69	5	0.34	5.41	0.0977
Pure error		0.19	3	0.063		

**Statistically significant at 99% of probability level.

X_1_: glucose concentration (g/L); X_2_: ammonium tartrate concentration (g/L); X_3_: harvesting time (h). *X*_1_^2^, *X*_2_^2^, and *X*_3_^2^: the quadratic terms; X_1_X_2_, X_1_X_3_, and X_2_X_3_: the interaction terms.

*R* ^2^ = 0.9968.

**Table 6:** Analysis of variance for the second-order polynomial model of GLA production by *Cuninghamella bainieri* 2A1 using repeated batch cultivation at 30°C for 96 h.

Source	Polynomial coefficients	Sum of squares	DF	Mean square	*F* value	Prob > *F*
Model		0.15	9	0.017	8.44	0.0031∗∗
Intercept	0.18					
*X*_1_	−0.047	0.022	1	0.022	11.05	0.0105∗
*X*_2_	0.025	6.250*E* − 003	1	6.250*E* − 003	3.13	0.1150
*X*_3_	−0.022	4.840*E* − 003	1	4.840*E* − 003	2.42	0.1584
*X*_1_^2^	7.976*E* − 003	1.724*E* − 004	1	1.724*E* − 004	0.086	0.7765
*X*_2_^2^	−0.012	3.917*E* − 004	1	3.917*E* − 004	0.20	0.6698
*X*_3_^2^	0.13	0.048	1	0.048	23.96	0.0012∗∗
*X*_1_ *X*_2_	0.071	0.041	1	0.041	20.31	0.0020∗∗
*X*_1_ *X*_3_	−3.750*E* − 003	1.125*E* − 004	1	1.125*E* − 004	0.056	0.8185
*X*_2_ *X*_3_	−0.014	1.513*E* − 003	1	1.513*E* − 003	0.76	0.4096
Residuals		0.016	8	1.999*E* − 003		
Lack of fit		0.014	5	2.824*E* − 003	4.52	0.1222
Pure error		1.875*E* − 003	3	6.250*E* − 004		

*Statistically significant at 95% probability level.

**Statistically significant at 99% of probability level.

*X*_1_: glucose concentration (g/L); *X*_2_: ammonium tartrate concentration (g/L); *X*_3_: harvesting time (h). *X*_1_^2^, *X*_2_^2^, and *X*_3_^2^: the quadratic terms; *X*_1_ *X*_2_, *X*_1_ *X*_3_, and *X*_2_ *X*_3_: the interaction terms.

*R* ^2^ = 0.9047.

**Table 7:** Fatty acid compositions of cellular lipid produced by *Cuninghamella bainieri* 2A1 in different repeated batch culture runs according to CCD.

Run	*X*_1_	*X*_2_	*X*_3_	^Δ6,9,12^C18:3	^Δ9,12^C18:2	^Δ9^C18:1	C18:0	C16:0
1	0	0	+1	0.33	0.98	2.04	1.26	1.06
2	+1	−1	−1	0.17	0.31	0.9	0.37	0.38
3	+1	−1	+1	0.15	0.87	1.56	0.91	0.82
4	0	0	0	0.21	0.47	1.48	0.9	0.72
5	0	+1	0	0.22	0.59	1.53	0.93	0.72
6	−1	−1	−1	0.45	0.32	0.94	0.38	0.41
7	+1	+1	−1	0.40	0.38	1.21	0.7	1.63
8	−1	0	0	0.18	0.16	1.77	0.87	0.8
9	0	0	−1	0.30	0.25	0.76	0.32	0.36
10	0	0	0	0.19	0.50	1.38	0.90	0.70
11	0	0	0	0.15	0.46	0.9	0.98	0.78
12	−1	−1	+1	0.40	0.78	2.31	1.0	1.04
13	0	0	0	0.18	0.55	1.3	0.88	0.6
14	+1	+1	+1	0.28	0.92	1.69	1.06	0.93
15	−1	+1	−1	0.35	0.28	0.92	0.34	0.39
16	0	−1	0	0.12	0.41	1.62	1.04	0.83
17	+1	0	0	0.20	0.54	1.51	0.87	0.7
18	−1	+1	+1	0.29	0.11	2.36	1.13	1.17

*X*_1_: glucose (g/L); *X*_2_: ammonium tartrate (g/L); *X*_3_: harvesting time (h); ^Δ6,9,12^C18:3: gamma-linolenic acid (g/L); ^Δ9,12^C18:2: linoleic acid (g/L); ^Δ9^C18:1: oleic acid (g/L); C18:0: stearic acid (g/L); C16:0: palmitic acid (g/L).

**Table 8:** The production of lipid and GLA by oleaginous zygomycetes using different substrates.

Microorganism	Substrate	Lipid (g/L)	GLA (mg/L)	Reference
*Mortierella isabellina *	Starch	3.7	181.3	[7]
*Cunninghamella echinulata *	Xylose	6.7	1119	[8]
*Cunninghamella echinulata* CCF-103	Corn steep	1.76	274	[15]
*Cunninghamella echinulata* ATHUM 4411	Tomato waste hydrolysate	3.58	802	[15]
*Mortierella isabellina *	Rice hulls	3.6	N^a^	[20]
*Cunninghamella echinulata *	Tomato waste hydrolysate	7.8	295.59	[22]
*Thamnidium elegans *	Glucose	15	1014	[26]
*Mortierella isabellina *	Cheese whey	8.1	301	[16]
*Zygorhynchus moelleri* BPIC 1703	Glucose	1.69	203	[27]
*Cuninghamella bainieri* 2A1	Glucose	6.2	400	This study

N^a^: not available.
